# Editorial: Tendinopathy Research- How Cutting Edge Developments Inform the Future Therapeutic Landscape

**DOI:** 10.3389/fbioe.2022.928144

**Published:** 2022-05-13

**Authors:** Stephanie G. Dakin, Alayna E. Loiselle, Andreas Traweger

**Affiliations:** ^1^ Nuffield Department of Orthopaedics, Rheumatology and Musculoskeletal Sciences (NDORMS), University of Oxford, Oxford, United Kingdom; ^2^ Center for Musculoskeletal Research, University of Rochester Medical Center, Rochester, NY, United States; ^3^ Institute of Tendon and Bone Regeneration, Spinal Cord Injury and Tissue Regeneration Centre Salzburg, Paracelsus Medical University, Salzburg, Austria

**Keywords:** tendon, tendinopathy, inflammation, biomaterials, tendon repair, topographical cues

There is a growing socio-economic need for effective and reproducible strategies to repair musculoskeletal tissue in general. In particular, acute tendon injury and chronic tendinopathies remain clinically challenging and novel treatment modalities are urgently needed. A low number of metabolically inactive cells combined with the avascular nature of tendons delays healing, whilst the innate reparative processes are incomplete and often associated with the formation of scar tissue compromising mechanical function. Therefore, novel treatments need to break the limits of tendon regeneration by harnessing novel cell instructive biomaterials and immunomodulatory, pro-regenerative therapeutic agents. To establish the current state-of-the-art, we recently undertook a special issue of Frontiers in Bioengineering and Biotechnology. This issue encompasses six articles that span from fundamental *in vitro* studies to molecular interrogation of human samples of tendinopathy. Amongst these articles, inflammation was identified as a key unifying theme, highlighting the ongoing importance of this process as a key driver of tendon cell fate, function and reparative capacity. Below, we briefly summarize the articles in this collection.

In their review article “*Reparative and Maladaptive Inflammation in Tendon Healin*g”, Arvind and Huang highlight the interesting paradigm of pathologic inflammation in the context of tendinopathy versus the reparative inflammation that is required to initiate a physiological healing process. The article summarizes important evidence for the therapeutic efficacy of immunomodulatory therapies and identifies the key gaps in knowledge, such as the optimal timing of these treatments, that must be addressed before translational success is achieved.

In the article “*Changes in physiological tendon substrate stiffness have moderate effects on tendon-derived cell growth and immune cell activation*”, Konar et al., examine the impact of substrate stiffness, as a surrogate for changes in the native vs diseased tendon extracellular matrix environments, on tendon cell and macrophage function. This study demonstrated the initiation of a pro-inflammatory macrophage phenotype on substrates of non-physiological stiffness, thereby identifying matrix changes as a potential mediator of altered macrophage function.

In the article “*Human Tendon Stem/Progenitor Cell (TSPC) Features and Functionality are Highly influenced by In Vitro Culture Conditions*”, Orfei et al. determine how modulating culture density influences the phenotypic, transcriptional, and secretory profiles of TSPCs. Their study demonstrates that TSPC’s are highly plastic in their response to external stimuli and that modulation of the cell culture density influences the identity of these cells *in vitro*. Their findings confirm the heterogeneity of TSPC subpopulations and identify cells grown at lower density favour TSPC development, which can be utilized for developing targeted regenerative medicine therapies for tendon disorders.

In their article “*Magnetic Nanoparticle-Mediated Orientation of Collagen Hydrogels for Engineering of Tendon-Mimetic Constructs*”, Wright et al., develop a convenient, non-invasive protocol to control the architecture of collagen I hydrogels. They achieve this using magnetic nanoparticles (MNP) and a magnetic field to induce remotely aligned or randomly organised collagen. They observed that tendon-like cells organized into parallel unidirectionally aligned collagen fibers and polydirectionally in non-aligned collagen constructs emulate physiological and pathological tendon niches respectively. These novel findings inform tissue engineering and organ-on-a-chip approaches for remotely controlling collagen matrix organization to recapitulate the native tendon.

Baldwin et al. investigate the influence of microscale topographical cues on tendon cell behaviour using electrospun polydioxanone scaffolds. In their original research paper “*Electrospun Scaffold Micro-Architecture Induces an Activated Transcriptional Phenotype within Tendon Fibroblasts*” the authors demonstrate by bulk RNA sequencing analysis that variable anisotropies yielded differential transcriptional responses mainly for large fibre diameter (≥2000 nm) scaffolds and fibroblast activation marker expression was reduced after culture on aligned scaffolds. These results confirm that specific scaffold architectures can be utilized to modulate stromal activation, and thus improve functional repair of tendon defects.

In their study “*Interleukin-17 Cytokines and Receptors: Potential Amplifiers of Tendon Inflammation*” Mimpen et al. delve into the role of Il-17 cytokine family members in tendon disease. They provide evidence that in comparison to IL-17RA, the IL-17RC receptor variant is more abundantly expressed in both, healthy and diseased tendon fibroblasts. They further confirm, that IL-17A is the most potent activator of NF-kB-mediated inflammation *in vitro*, whereas interestingly IL-17A, IL-17AF, and IL-17F all elicit a robust inflammatory response if co-administered with TNF-α. These results suggest IL-17 cytokines to act as inflammation amplifiers in tendinopathy.

The articles presented in this Research Topic demonstrate exciting progress in the field of tendon research, providing further insight into the role of inflammation in tendon pathogenesis and how biomaterials can be tuned to limit disease progression or improve tissue repair.

